# Spatial assessment of soil erosion using GIS-integrated RUSLE in the Wolaita zone, Ethiopia

**DOI:** 10.1371/journal.pone.0350986

**Published:** 2026-08-07

**Authors:** Selemon Thomas Fakana, Fekadu Fanjana Falta, Abera Abiyo Dofee

**Affiliations:** 1 Department of Environmental Science, College of Agriculture and Natural Resources, Gambella University, Gambella, Ethiopia; 2 Department of Natural Resources Management, College of Agriculture, Wolaita Sodo University, Wolaita Sodo, Ethiopia; 3 Department of Geography, College of Social Science and Humanities, Wachemo University, Hossana, Ethiopia; Escola de Engenharia de São Carlos da Universidade de São Paulo: Universidade de Sao Paulo Escola de Engenharia de Sao Carlos, BRAZIL

## Abstract

Soil requires focused management to ensure long-term productivity and ecological balance. The Revised Universal Soil Loss Equation (RUSLE) is an effective empirical model that, when combined with Remote Sensing (RS) techniques and Geographical Information Systems (GIS), enables the analysis of annual soil loss by integrating RUSLE factors and indices within the ArcGIS environment. In the current study region, limited attempts have been made to assess annual soil loss rates, identify erosion hotspots, and provide spatially explicit evidence to guide soil and water conservation measures. However, most inhabitants heavily depend on agriculture for survival. The RUSLE model was applied, integrating GIS and RS, to understand erosion rates and the spatial distribution of erosion intensity. The input datasets were acquired from diverse sources, including NASA POWER, FAO DSMW, USGS Earth Explorer, and the European Space Agency (ESA) LULC, and analyzed using ArcGIS 10.8.2 software. The finding unveiled that about 43,890.3 ha (9.77%), 30,388.14 ha (6.76%), and 1,459.17 ha (0.32%) of the study region experience high, very high, and severe soil erosion intensity, which requires effective conservation and management practices, including contour farming, terracing, check dams, vegetative barriers, crop rotation, mulching, agroforestry systems, and participatory watershed management. The annual soil erosion rate extends from 0.18 to 486 tons ha^-1^ year^-1^, with higher erosion intensity (46.09 to 486 tons ha^-1^ year^-1^) observed in the western portion of the study region. These areas are characterized by low vegetation cover, receiving higher rainfall, and a lack of effective support practices and cover management mechanisms. The findings offer insights into local erosion risk hotspots and support national initiatives in sustainable watershed management, climate resilience, and food security. Land managers, experts, and decision-makers can utilize the study results to develop the most effective long-term soil conservation and restoration plans.

## Introduction

Soils are essential natural resources that deliver critical ecosystem services to meet human needs [1]. Sustainable soil use depends on effective management to ensure long-term productivity and ecological balance [2]. Soil erosion refers to the accelerated removal of topsoil due to wind, water runoff, and agricultural practices such as tillage [3–5]. Various natural factors and anthropogenic pressures are driving forces for topsoil susceptibility to erosion [6,7], resulting in land productivity decline, depletion in soil water holding capacity, river siltation, soil nutrient loss, and water pollution [8]. Soil erosion is one of the significant environmental risks [9] that is anticipated to rise as a result of the diffuse effects of climate change [10] by amplifying rainfall erosivity, extreme weather events, reducing protective vegetation, and destabilizing soils. Globally, soil erosion is thought to be responsible for 85% of land degradation, causing a decline in crop yield up to 17% [11]. Pal and Chakrabortty [12] indicated that within the previous 150 years, around half of the topsoil on Earth has been lost. Globally, soil erosion and related factors result in the annual loss of more than 6 million hectares of productive land [13]. In Ethiopia, gully erosion still poses a threat to approximately 7.6 million hectares of agriculturally sustaining vertisols [14]. In the Ethiopian highlands, it is estimated that 1.5 billion tons of soil are eroded annually [15].

Numerous indices and indicators should be included in the framework for assessing soil erosion [16]. Planning and conserving soil and water resources depend heavily on it [17–19]. Soil erosion is estimated using the RUSLE model at the watershed basin scale [20,21]. An empirical model, the RUSLE, helps in estimating the average annual soil loss from sheet and rill erosion on cultivated or grassed slopes [22]. It comprises five factors, namely the conservation practices factor (P-factor), slope length and steepness factor (LS-factor), cover-management factor (C-factor), soil erodibility factor (K-factor), and rainfall erosivity factor (R-factor) [23].

The RUSLE is an empirical soil loss assessment model designed to account for statistical associations between erosion factors and measured soil erosion rates [24]. Due to its versatility and low data requirements [25], the RUSLE technique is applicable in a variety of settings and environments [26]. Globally, the USLE and RUSLE are among the most widely used soil erosion models, with 13.9% and 17.1% of applications, respectively [27]. The RUSLE model provides an insightful and efficient technique for mapping the spatial distribution of soil erosion by combining GIS and RS [28]. Remote sensing offers a reliable means for assessing soil erosion over large areas, both in time-series and synoptic contexts [29]. Many scholars have utilized the RUSLE model to estimate soil erosion, including studies in Poland [30], the Mediterranean region [31,32], Romania [33], Finland [34], Ethiopia [35,36], and even on a global scale [37].

To date, there have been few attempts to use RUSLE, GIS, and remote sensing methods to evaluate soil erosion in the current study region, despite the fact that most inhabitants rely primarily on agriculture for their livelihood. Wolaita Zone is one of the densely populated, agriculturally productive, and economically significant areas in Ethiopia, where the population depends on farming and livestock rearing for their livelihoods. Significant fruit trees grown include avocado, mango, banana, casimiroa, and peach. Its diverse agroecological conditions support a wide range of crops, making it a major producer of root and tuber crops, particularly enset, sweet potato, maize, teff, sorghum, and haricot bean, which are crucial for regional food security. It also supports substantial production of cereals, pulses, vegetables, and cash crops (coffee and chat/khat), contributing to both subsistence livelihoods and local markets [38,39]. Despite challenges such as soil erosion, degradation, and population pressure, the Wolaita Zone remains a key agricultural hub in the region, where intensive land use and mixed crop–livestock systems underpin the local economy. It is imperative that soil and water conservation measures be implemented, particularly in areas highly susceptible to soil erosion [40]. In the present study area, soil erosion monitoring is not conducted regularly to assess the extent of vulnerable regions and their environmental impact. The zone represents convergence of biophysical and socio-environmental stressors, characterized by high, spatially variable rainfall, steep, dissected terrain, and diverse agroecological zones, which together intensify soil erosion susceptibility. These natural vulnerabilities are further compounded by high population pressure, intensive land cultivation, and rapid land use change, as well as the zone’s hydrological connectivity to major downstream water infrastructure, notably the Gilgel Gibe III Dam and Lake Abaya. It has received limited attention, despite its high susceptibility to land degradation and its importance for local livelihoods. Despite the widespread application of RUSLE-based approaches, a critical gap persists in spatially explicit soil erosion assessment in the Wolaita Zone. While previous studies in Ethiopia have quantified soil erosion at watershed scales, few have explicitly linked erosion dynamics to downstream infrastructure vulnerability and landscape-level management priorities. Therefore, this study aims to bridge this gap by integrating GIS and remote sensing with the RUSLE model to quantify soil erosion risk, identify spatial hotspots, and provide a science-based foundation for targeted watershed management interventions. The findings offer insights into local erosion risk hotspots and support national initiatives in sustainable watershed management, climate resilience, and food security. Land managers, experts, and decision-makers can then utilize the study’s findings to develop effective strategies for long-term soil conservation and restoration.

## Materials and methods

### Study area

**Fig 1:**
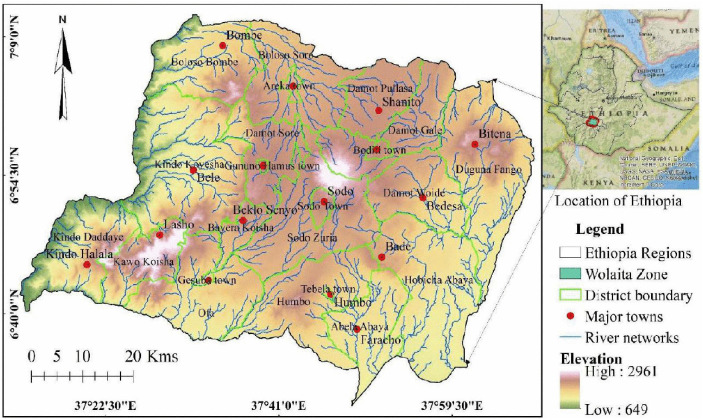
Map of the study area. Source: HDX-OCHA (https://data.humdata.org/) and USGS SRTM (https://earthexplorer.usgs.gov/).

The Wolaita Zone is situated between 37^0^13’23“to 38^0^’8’10” E longitude and 6^0^30’23” to 7^0^11’29” N latitude, encompassing approximately 4509 km^2^. It lies about 300 km south of Addis Ababa, Ethiopia’s capital. It is structured into more than sixteen districts (Fig 1). It has an elevation range of 649–2961 m.a.s.l., with slope gradients of 0–73°, experiencing a bimodal rainfall dynamic, with the forty-year mean annual rainfall ranging from 2133 to 2463 mm. The zone has Eutric Nitosols (Ne), Haplic Xerosols (Xh), Plinthic Ferralsols (Fp), Eutric Cambisols (Be), and Ochric Andosols (To) soil types. The eastern portion of the study region is dominated by Haplic Xerosols (Xh), whereas the western portion is described by Eutric Cambisols (Be). The zone is characterized by clay, clay loam, sandy clay loam, loam, and sandy clay soil textures [41,42].

### Data sources

**Fig 2:**
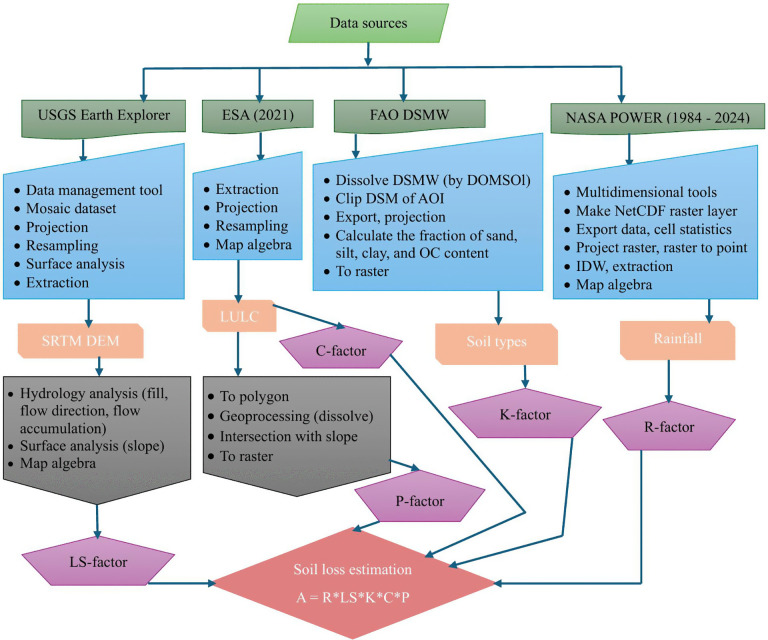
Schematic diagram illustrating the methodological workflow.

Input datasets (meteorological data, soil properties, topography, satellite images, and LULC data) were accessed from several open-access datasets due to their reliability, accessibility, and suitability for regional-scale soil erosion modeling in data-scarce environments (Table 1). To analyze the erosivity factor, precipitation data were retrieved from NASA POWER in NetCDF format. NASA POWER provides long-term, quality-controlled, and spatially consistent gridded climate data, though it may not fully capture localized rainfall variability caused by topographic and microclimatic differences. The soil type data were acquired from the FAO/UNESCO Digital Soil Map of the World (DSMW) in ESRI shapefile format. The FAO DSMW offers harmonized global soil information compiled from various national and regional surveys, and was chosen for its standardized classification, global consistency, and suitability for deriving soil erodibility (K-factor) in RUSLE modeling. The percentages of silt, sand, clay, and organic carbon in the topsoil were also determined for each soil type in the study area. For instance, the percentages of sand, silt, clay, and organic carbon for eutric cambisols (Be) in the study area were 36.4%, 37.2%, 26.4%, and 1.07%, respectively. The SRTM DEM was accessed from USGS Earth Explorer for path 169 and row 55, and a mosaic dataset was generated using data management tools. We processed the Land Use and Land Cover (LULC) dataset from the European Space Agency (ESA) WorldCover dataset V200, which has a 10 m resolution. It offers recent, globally consistent, and high-resolution data derived from Sentinel-1 and Sentinel-2 imagery, ensuring reliability and comparability. All input datasets were standardized by converting them to raster format, projecting them to the WGS 1984 UTM Zone 37N coordinate system, and resampling them to a uniform 30 m spatial resolution. The Ethiopian administrative boundaries and area of interest (AOI) shapefiles were accessed and processed from the Humanitarian Data Exchange (HDX-OCHA) data portal. The overall diagrammatic illustration of the methodology framework is presented in Fig 2.

**Table 1:** RUSLE factors and data sources used for soil erosion analysis.

RUSLE factors	Data	Data type	Spatial resolution	Resampled resolution	Source
R-factor	Rainfall	NetCDF	0.5	30	NASA POWER (https://power.larc.nasa.gov/)
K-factor	Soil	shapefile	Polygon	30	FAO DSMW (https://www.fao.org/)
LS-factor	Slope length & steepness	TIF	0.00027	30	SRTM DEM (https://earthexplorer.usgs.gov/)
C-factor	LULC	TIF	10	30	ESA (https://esa-worldcover.org/en)
P-factor	Soil conservation practices	TIF	30	30	ESA (https://esa-worldcover.org/en) and SRTM DEM

### Data analysis

The annual rates of soil erosion were estimated using the Revised Universal Soil Loss Equation (RUSLE) model [43]. It is the most popular empirical model for determining the average yearly soil loss per unit area [44], calculated by a simple mathematical expression (Eq. 1). Its integration with GIS enables the spatially explicit quantification and mapping of soil loss, particularly in large and data-scarce regions, making it a cost-effective and replicable tool.


$$\begin{array}{c} 𝐀=𝐑*𝐊*𝐋𝐒*𝐂*𝐏 \end{array}$$ (1)

Annual estimated erosion rate (tons ha^-1^ year^-1^) is denoted as A, rainfall erosivity factor (MJ mm ha^-1^ h^-1^ year^-1^) is given by R, soil erodibility factor (t ha h h^-1^ MJ^-1^ mm^-1^) is represented by K, slope length factor is represented by L, slope steepness factor is given in S, cover-management factor is defined by C, support practice factor is designated by P.

Using multidimensional tools in the ArcGIS interface, a NetCDF raster precipitation layer was created, exported, and projected to the WGS 1984 UTM Zone 37N coordinate system. Using the Cell Statistics function under Spatial Analyst Tools, a gridded layer was generated and converted into a point precipitation layer. The inverse distance weighted (IDW) approach (using grid codes) was used to interpolate a 40-year (1984–2024) mean annual rainfall dataset from NASA POWER, with a 30 m cell resolution (S2_NASA POWER precipitation). Using IDW to interpolate rainfall erosivity can introduce unrealistic gradients, particularly in heterogeneous terrain or areas with sparse meteorological stations. To mitigate the coarse resolution of gridded rainfall data, a 40-year mean annual rainfall from NASA POWER was interpolated to better represent spatial variability and reduce interannual noise. The interpolated R-factor was cross-validated against observed rainfall trends and R-values from similar regional studies reported in the literature [45–48]. Residual uncertainties are acknowledged, and the results are interpreted primarily in terms of relative spatial patterns rather than absolute values. Hurni’s empirical equation [49], calibrated for Ethiopia (Eq. 2), was used to determine the R-value.


$$\begin{array}{c} 𝐑=-8.12+(0.562*𝐏) \end{array}$$ (2)

P is the mean annual rainfall (mm/year), and R is the rainfall erosivity factor in MJ/mm/ha/year,

The K-factor quantifies how sensitive soil particles are to detachment and movement caused by rainfall and runoff effects [50]. It is determined by considering the soil’s chemical and physical properties, including its permeability, texture, organic matter content, and structure [51, 52]. K-values range from 0 to 1, where 0 indicates the soil has the minimum vulnerability to erosion. Based on soil type in the study region, the sand, silt, clay, and organic carbon contents (%) were matched to soil types in the ArcGIS interface using the “joins” and “relates” functions. Then, the fraction of the topsoil (%) for silt, sand, clay, and organic carbon was calculated (Eq. 3), and K-values were determined employing the model proposed by [53]. Waterbodies are assigned a K-value of 0, since there is no exposed soil in waterbodies, and they do not cause soil loss via rainfall-induced detachment or runoff.


$$\begin{array}{c} K=\left\{0.2+0.3e^{\left[-0.0256SAN\left(1-SIL/100\right)\right]}\right\}*({\frac{SIL}{CLA+SIL})}^{0.3}*\left[1-\frac{0.25C}{C+e^{\left(3.72-2.95C\right)}}\right]*\left[1-\frac{0.7SN}{SN+e^{22.9SN-5.51}}\right] \end{array}$$ (3)

SAN, SIL, and CLA denote the soil sand, silt, and clay contents (%), respectively; the soil organic carbon content (%) is represented by C; SN = 1 − SAN/100.

The topographic factor, which measures the impact of topography on soil erosion, was derived from SRTM-DEM with a 30-m spatial resolution. The SRTM DEM for path/row 169/055 was retrieved from the USGS EarthExplorer, and the TIF files (n06_e037_1arc_v3, n06_e038_1arc_v3, n07_e037_1arc_v3, and n07_e038_1arc_v3) were imported into ArcGIS. Using data management tools, the individual tiles were compiled into a mosaic dataset, as a single raster, projected to the WGS 1984 UTM Zone 37N coordinate system, and resampled to 30-m spatial resolution. Then, using the spatial analyst tool, the Area of Interest (AOI) was extracted. After this, DEM fill was processed to fill sinks and imperfections in the hydrological analysis, and flow accumulation was generated. The slope (^o^) was calculated using surface analysis, and the raster calculator was used to determine the LS factor. The slope-length (L) factor indicates the proportion of soil loss from the field slope length λ (m) to that from a 22.13 m length under the identical condition. Slope steepness (S) is defined as the ratio of soil loss from the field slope gradient (%) to that from a 9% slope under identical conditions [54]. The LS factor indicates that erosion rises with slope length and angle, calculated using Equation (4).


$$\begin{array}{c} LS=({\frac{A}{22.13})}^{0.4}({\frac{sin\beta }{0.0896})}^{1.3} \end{array}$$ (4)

Where LS is the combined slope length and slope steepness factor, A denotes an upslope contributing area, and a degree slope is given by β.

The C-factor illustrates how soil loss is influenced by crop management, land cover, and management techniques in comparison to bare regions [55]. It has a value between 1 and 0, where 1 denotes no cover, and a value close to 0 represents a well-vegetated area [56]. The C-factors are determined by LULC type. Given the limited availability of region-specific field measurements, the C-values were selected from the literature reporting C-factors for comparable land-use types in Ethiopia, considering observed local soil and water conservation practices and consultations with zonal land management guidelines to ensure contextual relevance (Table 2). Waterbodies were assigned a C-factor of 0 because they are considered non-erodible surfaces. The C-factor of 0 ensures that these areas do not contribute to sediment yield in the RUSLE calculation. A typical C-value for herbaceous wetland areas extends from 0.001 to 0.05. The present study utilizes C-values of 0.03, as the area is characterized by vegetation cover, high soil moisture, and low surface runoff, which significantly reduces erosion. TheThe efficiency of soil conservation techniques in lowering erosion is shown by the P-factor. Under specific practice settings, tillage upslope and downslope produce a ratio of soil loss [43]. It takes into account soil conservation techniques, such as terracing, contour plowing, bunds, and other erosion-control measures, implemented in a particular region [65–67]. The ESA Worldcover 10m V200 LULC map was resampled to 30 m resolution, converted to a polygon, dissolved by grid code, and intersected with slope gradients (%) in ArcGIS software. The P-factors are assigned based on LULC types and slope gradients. Due to the limited availability of detailed field-based data on local soil and water conservation practices, the P-values were inferred from generalized management recommendations (Table 3). This approach is widely applied in RUSLE-based studies in data-limited environments, where LULC and slope are used to approximate the effectiveness of conservation practices and land management conditions [45,68,70,72]. While this approach introduces some uncertainty, cross-checking with previous regional studies ensured their relevance. Therefore, soil erosion results are interpreted in terms of relative spatial patterns rather than absolute values. The P-values fall between 0–1; the stronger the surface protection mechanisms, the smaller the P-value. The bare land/sparse vegetation was assigned a P-value of 0.8 in accordance with the ESA WorldCover 10m V200 LULC classification, which combines bare land and sparse vegetation into a single class. Within the study area, such surfaces are rarely completely devoid of micro‑topographic roughness or scattered vegetation, both of which provide minimal resistance to runoff compared to perfectly bare soil. Thus, a value slightly below 1 was adopted to better reflect local conditions [71].

**Table 2:** C-value assignment for various LULC classes, Wolaita Zone, Ethiopia.

LULC types	C-value	Description	References
**Tree cover (dense forest)**	0.01	Continuous canopy cover minimizes raindrop impact and runoff	[43, 57, 58]
**Shrubland**	0.15	Moderately protective, with less canopy and ground cover than forests.	
**Grassland**	0.05	Dense herbaceous cover reduces erosion significantly.	[43, 57, 59]
**Agricultural land (Cropland)**	0.5	It varies according to crop type, tillage, and growth stage. Row crops without cover have a higher risk of erosion.	[43, 58]
**Built-up areas**	1	Impervious surfaces do not erode; instead, they increase runoff and contribute to downstream erosion.	[60]
**Bare land /sparse vegetation**	1	No vegetation cover; maximum erosion risk.	[58]
**Permanent waterbodies**	0	No soil erosion—often excluded or set as 0.	[61, 62, 63]
**Herbaceous wetland**	0.03	Saturated soil and dense cover result in negligible loss rates.	[63, 64]

**Table 3:** Different LULC types and adopted P-value assignments from published sources, considering local conditions.

Land-use types	Agricultural land [45,68,69]						Built-up areas [70]	Bare land/sparse vegetation [71]	Forest land	Grassland	Herbaceous wetland	shrubland	Waterbodies [48,70]
Slope (%)	0.5	5-10	10-20	20-30	30-50	>50	all	all	all	all	all	all	all
P-factor	0.1	0.12	0.14	0.19	0.25	0.33	1	0.8	0.5	0.9	0.8	1	0

## Results

### Erosivity factor

**Fig 3:**
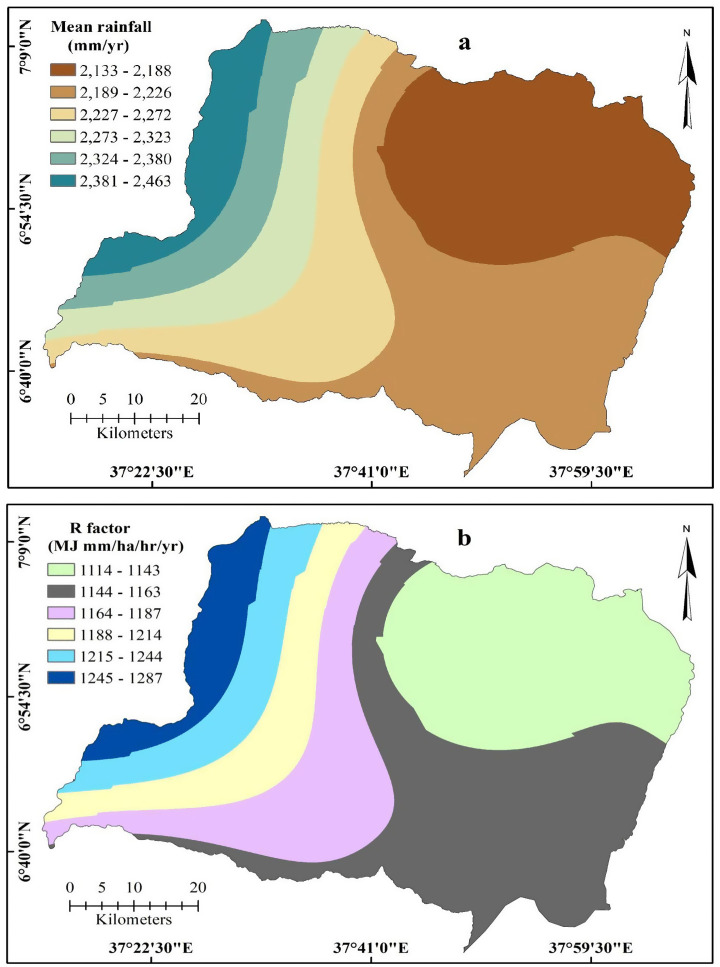
Rainfall erosivity a) Rainfall b) R-factor. Source: NASA POWER (https://power.larc.nasa.gov/).

Rainfall is a significant driving force of soil erosion [73]. In general, soil erosion increases in tandem with rainfall intensity due to increased runoff [24]. The R-factor reflects the erosive force of rainfall and quantifies the effect of raindrop impact, which initiates soil detachment and transport, and is influenced by rainfall intensity, kinetic energy, duration, and the frequency of intense storms. The eastern portion of the study region exhibited low erosivity, covering approximately 1,615.93 km² (58.23%) and with R-values ranging from 1114 to 1163. The western portion of the region of interest receives higher rainfall amounts (Fig 3a), resulting in a higher erosivity potential (R-values of 1245–1287 MJ mm ha^-1^ hr^-1^ yr^-1^), accounting for 6.83% (Fig 3b, Table 4). Overall, the R-value extends from 1114 to 1287 MJ mm ha^-1^ hr^-1^ yr^-1^, implying a positive association with rainfall distribution. Comparable findings were reported from the Rib watershed, Ethiopia (R-values ranged from 921 to 1468) [45], the Gumara watershed, Ethiopia (R-values 1013.45 to 1159.77) [46], and the Megech river catchment, Ethiopia (R-values 515–1850) [47], indicating a positive relationship with rainfall. It is also in line with a study conducted in the Irga watershed on the eastern edge of the Chota Nagpur Plateau, India, where the R-factor is positively correlated with rainfall and exhibits a spatial distribution pattern similar to that of rainfall [48].

**Table 4:** RUSLE factors, areal coverage, and erosion intensity, in the Wolaita Zone, Ethiopia.

RUSLE factors	Values	Area		Soil erosion intensity	RUSLE factors	Values	Area		Soil erosion intensity
		km^2^	%				km^2^	%	
R-factor	1114 - 1143	1192.52	26.65	Very low	C-factor	0	93.69	2.08	No
	1144 - 1163	1423.41	31.58	Low		0.001–0.0282	567.64	12.6	Very low
	1164 - 1187	722.07	16.01	Moderate		0.0283–0.0502	748.4	16.61	Low
	1188 - 1214	477.89	10.59	High		0.0503–0.151	1380.18	30.63	Moderate
	1215 - 1244	385.3	8.55	Very high		0.152–0.502	1673.51	37.14	High
	1245 - 1287	307.88	6.83	Severe		0.503–0.8	42.34	0.94	Very high
K-factor	0	27.8	0.62	No	P-factor	0	91.7	2.04	No
	0.0001 - 0.0193	45.44	1.01	Very low		0.0001 - 0.1176	743.83	16.54	Low
	0.0194 - 0.0196	907.62	20.13	Low		0.1177 - 0.251	945.77	21.02	Moderate
	0.0197 - 0.0208	1191.77	26.43	Moderate		0.2511 - 0.502	555.23	12.34	High
	0.0209 - 0.022	904.24	20.05	High		0.5021 - 1	2161.8	48.06	Very high
	0.0221 - 0.0223	1432.24	31.76	Very high					
LS-factor	0 - 5.67	3457.92	76.87	Very low					
	5.68 - 28.35	944.07	20.99	Low					
	28.36 - 79.4	84.92	1.89	Moderate					
	79.41 - 187.16	9.49	0.21	High					
	187.17 - 408.35	1.66	0.04	Very high					
	408.36 - 1451.92	0.27	0.01	Severe					

### Erodibility factor

**Fig 4:**
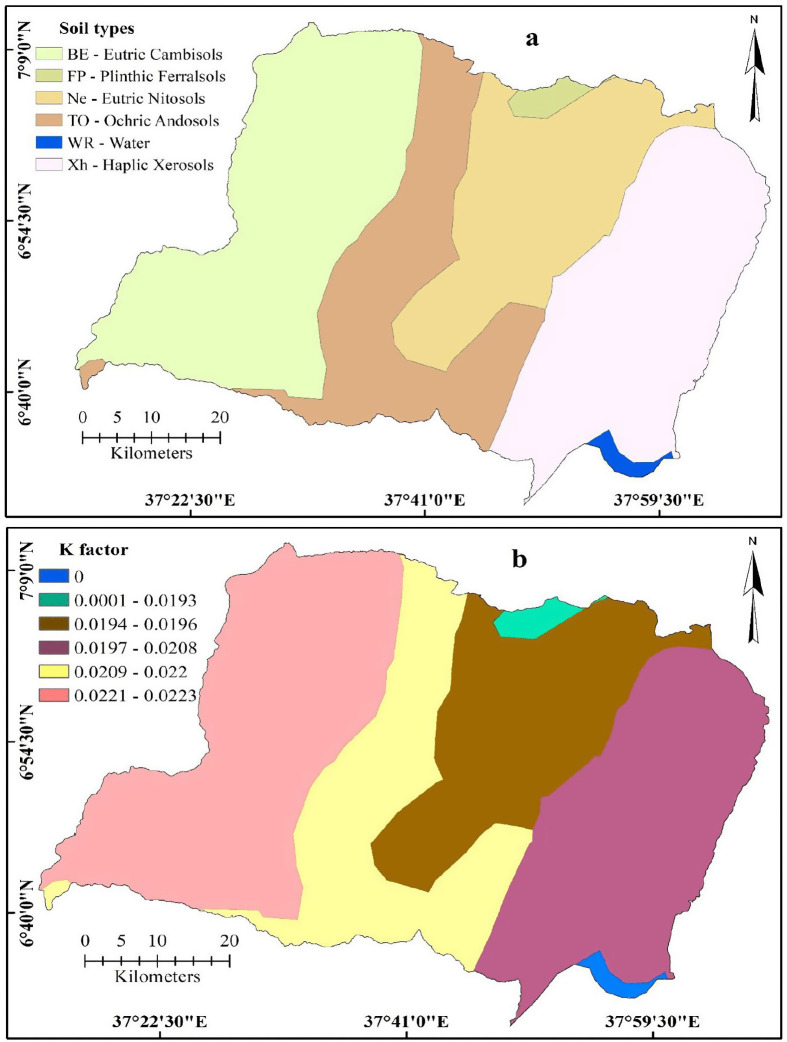
Soil erodibility a) soil types b) K-factor. Source: FAO DSMW (https://www.fao.org/soils-portal/data-hub/soil-maps-and-databases/faounesco-soil-map-of-the-world/en/).

The K-factor quantifies how easily soil can be eroded under standard rainfall, slope, and land cover conditions. It reflects the soil’s inherent properties, particularly organic matter, texture, permeability, and structure [43]. The finer and richer soil textures are less susceptible to erosion and more resilient to particle dissociation [36]. About 1432.24 km^2^ area of interest showed very high soil erodibility characteristics, with K-values (0.0221 to 0.0223). This is in line with findings from the Gidabo watershed in the Rift Valley Basin, Ethiopia, which indicate that higher k-values indicate greater susceptibility to soil erosion [68]. These are found in the western portion of the study region (Fig 4b), where they are represented by Eutric Cambisols (Be) (Fig 4a). These soils are highly erodible because they typically have shallow, weakly developed soils, low organic matter, and weak structure [43, 74]. The loss of fertile topsoil reduces crop productivity by depleting essential nutrients, increases input costs, and diminishes household income. Over time, declining soil fertility and land degradation threaten regional food production, exacerbating food insecurity.

The middle portion of the study zone is dominated by Ochric Andosols (To), which are also highly vulnerable to erosion due to their weak structure and low organic matter content in the surface horizons [58,75]. Haplic xerosols (Xh) are typically arid soils with low organic matter, coarse texture, and low water-holding capacity, and are moderately vulnerable to erosion [49]. These soils are observed in the eastern sites of the zone, covering an area of 1,191.77 km², with K-values ranging from 0.0197 to 0.0208 (Table 4). Due to its well-structured nature, clay-rich composition, high organic matter content, and good permeability, the Eutric Nitosols (Ne) soil type is characterized by low erodibility [49,61]. The Plinthic Ferrasols (Fp) are the least vulnerable to erosion due to their strongly weathered nature and resistance to detachment, resulting from a stable structure [76, 77]. The greater the erodibility value, the greater the erosion [78]. The lowest K-value and the lowest erodibility coincide with gentle-slope areas and low rainfall intensity, whereas the highest soil erodibility occurs at steep-slope regions.

### Topographic factor

**Fig 5:**
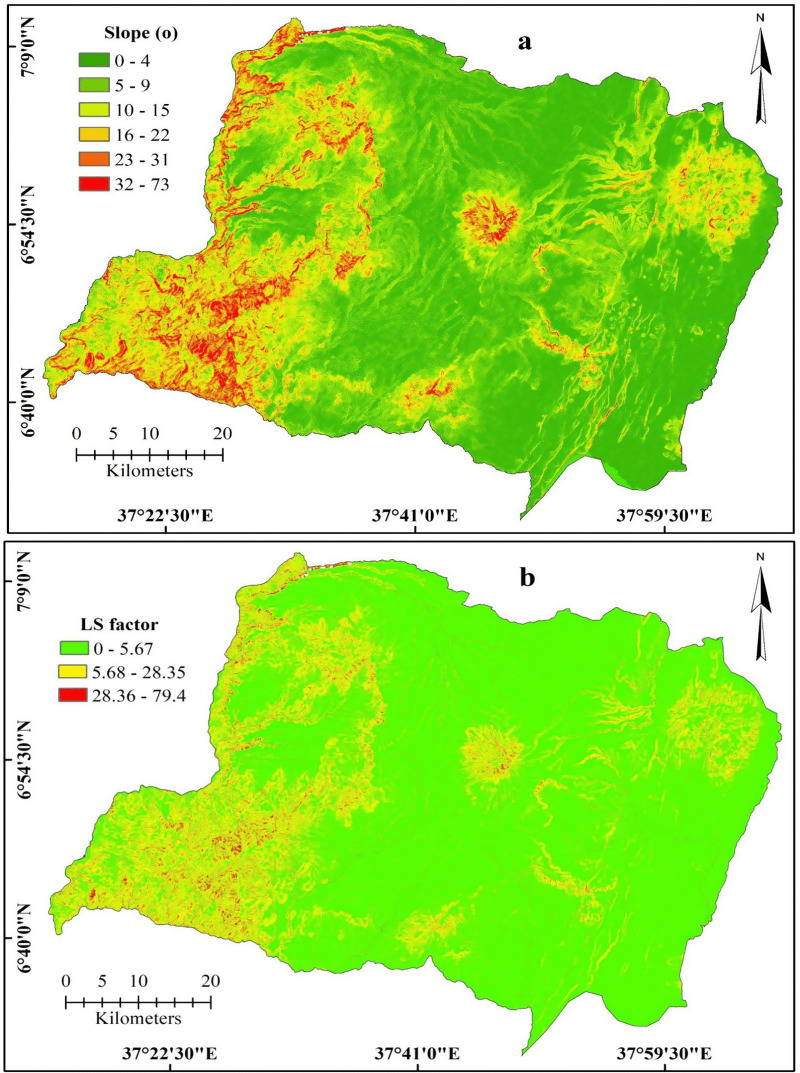
Topographic factor a) slope b) LS-factor. Source: USGS SRTM (https://earthexplorer.usgs.gov/).

The combined effects of slope steepness (S) and slope length (L) on soil erosion are represented by the LS factor, which plays a significant role because topography directly influences the velocity, volume, and erosive force of surface runoff. The result shows that the study region is illustrated by highly heterogeneous terrain with steeper and longer slopes (23 ° to 73 °), found in south-western, north-western, central (Damota mountain), and east-northern locations (Fig 5a). The majority of the study region encompassing an area of 3457.92 km^2^ (76.87%) (Table 4), found to be very low erosion intensity, with LS-values of 0 to 5.67 (Fig 5b). The longer and steeper the slope, the greater the rate of soil erosion [79,80]. Longer slopes (L) allow for greater runoff accumulation, increasing erosion potential; steeper slopes (S) increase runoff velocity, thereby enhancing water’s ability to isolate and transport soil particles. Al-Mamari et al. [81] also reported that the steepness and length of slopes enhance overland flow and reduce the concentration time, thereby intensifying soil erosion. The most erosion-prone regions contain steep hillslopes that are extensively cultivated due to land scarcity, combined with shallow soils and high rainfall erosivity, which magnifies the LS-factor values, resulting in disproportionately high erosion in these areas. However, the higher LS values (79.41–1451.92) occupy only a very limited portion of the landscape, 11.42 km² (0.26%) of the study area (Table 4) and therefore do not appear prominently at the map scale presented in Fig 5b. The total area of the study region seems to be reduced in the calculated LS-factor layer due to masking, assigning LS-values of 0, or clipping during preprocessing steps in the ArcGIS interface.

### Cover-management factor

**Fig 6:**
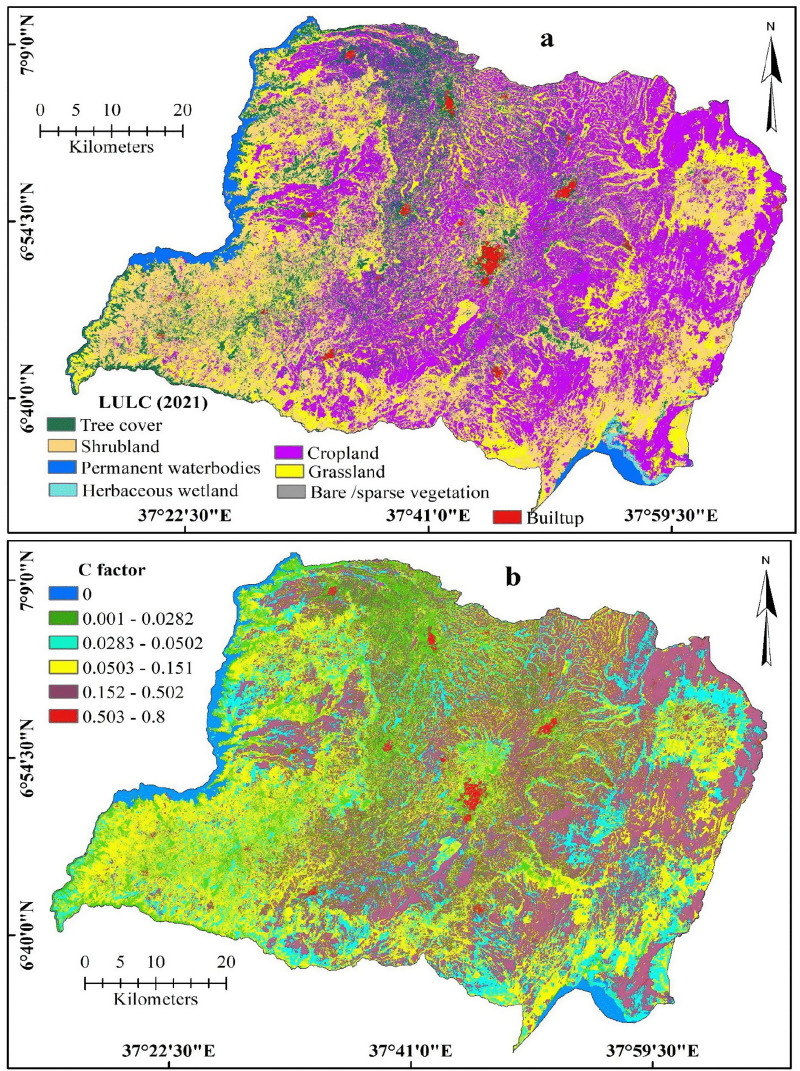
Cover management a) LULC b) C-factor. Source: ESA (https://esa-worldcover.org/en).

The C-factor is a vital variable in erosion modeling, representing the extent to which vegetation and land management practices reduce soil loss. The southwest regions of the area of interest exhibited dense and healthy vegetation cover, with C-values ranging from 0.001 to 0.0502 (Fig 6b), covering an area of 11316.04 km^2^ (29.21%) (Table 4), and experienced low erosion intensity. High-vegetation-cover regions are typically less likely to experience soil erosion [82]. Fig 6a shows that the western and southeastern margins of the region have no erosion risk, as they are covered by water bodies (Gilgel Gibe III dam, Omo River, and Lake Abaya) and herbaceous wetlands, totaling 98.69 km² (2.08%) (Table 4). The findings indicated that the majority of the study region, 1673.51 km² (37.14%), exhibited poor cover management practices, with C-values ranging from 0.15 to 0.502 (Table 4), making it susceptible to high erosion intensity, which necessitates prioritizing soil conservation efforts. High erosion often overlaps with regions experiencing significant vegetation pressure due to fuelwood collection, grazing intensity, and agricultural expansion. Reduced vegetative cover reduces soil protection during the rainy season, increasing erosion and sediment mobilization. The lower the C-factor, the greater the soil protection the cover offers, indicating the lower erosion intensity.

### Support practice factor

**Fig 7:**
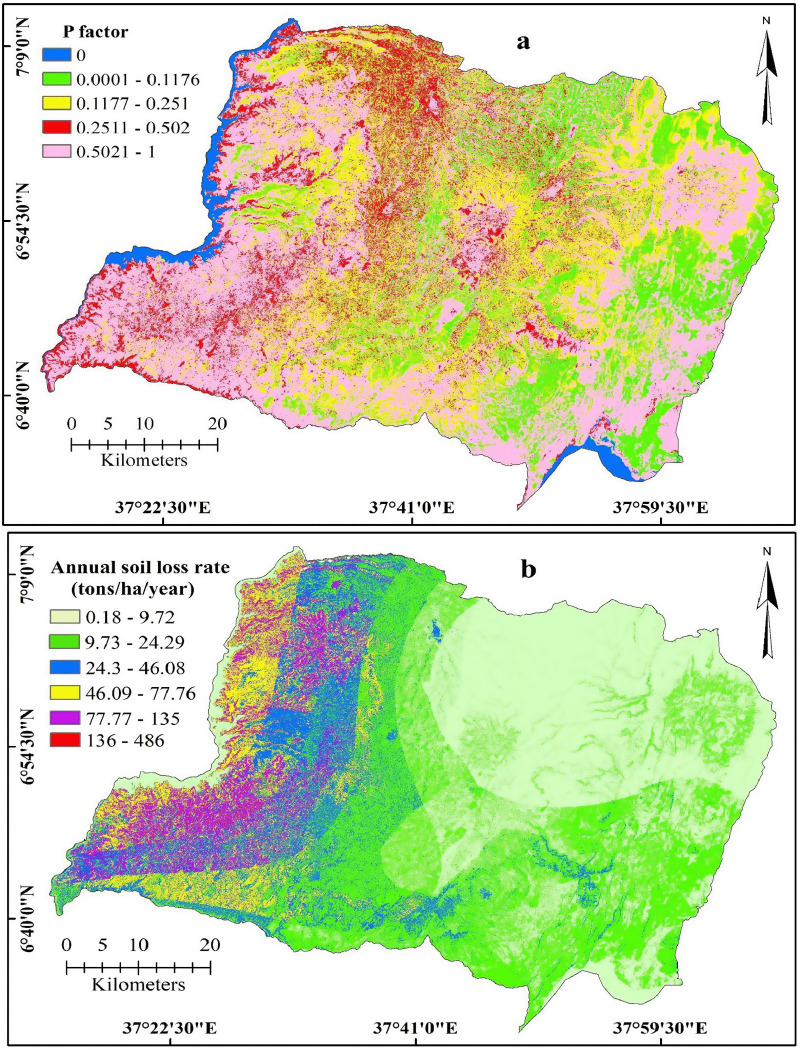
a) Support practice (P-factor) b) Wolaita Zone annual soil loss rate. Source; ESA (https://esa-worldcover.org/en) and USGS SRTM (https://earthexplorer.usgs.gov/).

Fig 6a describes the land use land cover of the area of interest. P-factor computes the effectiveness of land management mechanisms in reducing runoff and preventing soil loss. It illustrates how water and soil conservation efforts impact the rate of soil erosion [82]. The areas covered by vegetation, crops, grassland, and shrublands tend to have low P-values (0.001 to 0.1176) (Fig 7a), covering an area of 743.83 km^2^ (16.54%) (Table 4), and exhibit low erosion intensity. However, about 2161.8 km^2^ (48.06%) of the study region (southwestern, western, central, southeastern, and northeastern) experiences very high erosion intensity, having P-values (0.5021 to 1), requiring intensive conservation practices to reduce the volume and velocity of surface runoff, thereby minimizing soil erosion. These regions are described by steep slopes (23 ° to 73 °) (Fig 5a) and covered by bare land/sparse vegetation (Fig 6a). Furthermore, highly erodible areas correspond to zones where traditional plowing on steep slopes, limited contour farming, and inadequate soil conservation measures remain prevalent, resulting in reduced ground cover and increased surface runoff, thereby amplifying soil loss.

### Mapping erosion risk areas and prioritizing

The study revealed that the annual soil loss ranges from 0.18 to 486 tons ha^-1^ year^-1^, with a mean yearly soil loss of 24.69 tons ha^-1^ year^-1^. Comparable results were reported from different locations of Ethiopia: Gununo watershed (0–359.99 tons ha^-1^ year^-1^ with a mean annual soil loss of 22.31 tons ha^-1^ year^-1^) [83], Gumara watershed (0.01–442.92 tons ha^-1^ year^-1^ with mean annual soil loss of 42.67 tons ha^-1^ year^-1^) [46], Rib watershed (0–807 tons ha^-1^ year^-1^ with annual soil loss of 68 tons ha^-1^ year^-1^) [45], Beshillo catchement (0–935 tons ha^-1^ year^-1^ with mean annual soil loos of 37 tons ha^-1^ year^-1^) [84]**.** The western portion of the study zone (Fig 7b) exhibited high, very high, and severe erosion intensity, covering 43,890.3 ha (9.77%), 30,388.14 ha (6.76%), and 1,459.17 ha (0.32%), respectively (Table 5). These areas are characterized by low vegetation cover, barren land, receive high rainfall, have steeper, longer slope gradients, and are dominated by eutric cambisols (Be). This higher erosion intensity may disproportionately contribute to sediment load at the Gilgel Gibe III Dam (built on the Omo River), reducing reservoir storage capacity, shortening the dam’s lifespan, and increasing maintenance costs. It also has significant and detrimental effects on lakes (Lake Abaya) and riverbanks. Eroded soil from upstream areas can cause sedimentation, reduce water quality for downstream uses, alter hydrological processes, lead to flooding, algal blooms or eutrophication, degrade habitats for aquatic ecosystems, and incur economic costs. Catchment area management, including afforestation and reafforestation, crop rotation and mulching, agroforestry, contour farming and terracing, maintaining vegetated buffers, grass strip planting, and land use planning, are mechanisms to mitigate the negative impacts of soil erosion on the dam and downstream areas. Additionally, watershed-based planning, effective monitoring systems, updating land-use databases, community involvement, and participatory planning can help mitigate erosion risks. The middle, north, northeastern, and southeastern margins of the study regions showed low erosion intensity, with annual soil loss rates of 0.18 to 9.72 tons ha^-1^ year^-1^ (Fig 7b). These areas experience low erosivity, erodibility, C-factor, LS-factor, and support practice factors values.

**Table 5:** Annual soil erosion rate, area coverage, erosion intensity, and priority level.

Annual soil erosion rate (tons ha^-1^year^-1^)	Area		Soil erosion intensity	Priority level
	ha	%		
0.18 - 9.72	171,054.45	38.06	Very low	6^th^
9.73 - 24.29	139,116.6	30.96	Low	5^th^
24.3 - 46.08	63,491.49	14.13	Moderate	4^th^
46.09 - 77.76	43,890.3	9.77	High	3^rd^
77.77 - 135	30,388.14	6.76	Very high	2^nd^
136 - 486	1,459.17	0.32	Severe	1^st^

## Discussion

This study provides a robust spatial assessment of soil erosion dynamics in the Wolaita Zone using a GIS-integrated RUSLE framework, revealing pronounced spatial heterogeneity driven by interactions among climatic forcing, terrain characteristics, soil properties, and land management practices. The estimated mean annual soil loss (24.69 tons ha ⁻ ¹ year ⁻ ¹) is consistent with values reported for comparable Ethiopian highland watersheds [83,85–87], reinforcing the broader understanding that soil erosion remains a persistent and severe form of land degradation in intensively cultivated tropical highland systems. Rainfall erosivity emerged as a dominant control on erosion patterns, with elevated R-factor values reflecting the strong dependence of erosion processes on rainfall intensity and kinetic energy, which directly influence soil detachment and transport mechanisms. The observed positive correlation between rainfall distribution and erosivity is consistent with findings from other Ethiopian watersheds [45–47]. Importantly, this reinforces concerns that projected increases in rainfall variability and extreme events under climate change scenarios may further intensify erosion risks in already vulnerable landscapes. Thus, the identified erosion hotspots are not only current degradation zones but also likely future areas of risk amplification.

Soil erodibility further modulates this climatic influence, with highly erodible soils such as Eutric Cambisols and Ochric Andosols exhibiting greater susceptibility to detachment due to weak structural development and low organic matter content [88–90]. The spatial coincidence of high K-values with areas of severe erosion aligns with previous findings that soil intrinsic properties significantly govern erosion response under similar environmental conditions [68]. Conversely, the relative stability of Eutric Nitosols and Plinthic Ferralsols, attributed to their well-developed structure and higher clay content, underscores the importance of soil resilience in mitigating erosion processes [91–93]. These results highlight that erosion risk is not solely a function of external forcing but also of inherent soil characteristics, necessitating site-specific conservation strategies rather than uniform interventions. Topographic factors strongly influence erosion intensity, particularly in steep, dissected landscapes. The concentration of high erosion rates in areas with steep and elongated slopes confirms the critical role of terrain in accelerating runoff velocity and sediment transport capacity [94,95]. Although areas with extreme LS values occupy a relatively small spatial extent, their contribution to total sediment yield is disproportionately large, consistent with observations from similar mountainous environments [81]. This finding emphasizes the need for targeted management of slope-dominated landscapes, where even localized interventions can yield significant reductions in sediment exports.

Land cover and management practices represent key anthropogenic controls on erosion. The study demonstrates that areas with dense vegetation cover exhibit markedly lower erosion rates, confirming the well-established role of vegetation in intercepting rainfall, enhancing infiltration, and stabilizing soil through root systems [96–98]. In contrast, regions experiencing agricultural expansion, overgrazing, and vegetation depletion show elevated C-values and correspondingly higher erosion risks. This pattern aligns with global evidence linking land use change and vegetation degradation to accelerated soil erosion and ecosystem degradation [97,99,100]. Furthermore, the predominance of high P-factor values indicates limited implementation of effective soil and water conservation measures, particularly in steep agricultural areas. This reflects systemic challenges in the Ethiopian highlands, where socio-economic pressures and traditional farming practices constrain the adoption of conservation technologies [101].

Beyond the individual influence of each RUSLE parameter, the identification of erosion hotspots in the western portion of the Wolaita Zone highlights the cumulative effect of unfavorable biophysical and management conditions. The spatial pattern of severe erosion zones is explained by the synergistic interaction of multiple factors, including high rainfall erosivity with steep and elongated slopes, and degraded vegetation cover. These areas not only experience high soil loss but also serve as critical sediment source zones with downstream implications. The potential sedimentation from these areas in major water bodies, such as the Gilgel Gibe III Dam and Lake Abaya, underscores the broader environmental and economic consequences of upstream land degradation [97,98]. Sediment accumulation in reservoirs reduces storage capacity, alters hydrological regimes, and increases maintenance costs, thereby linking local land management practices to regional water resource sustainability [99]. Similar interactions have been reported in other Ethiopian highland systems, where the co-occurrence of steep terrain, high rainfall, and land degradation significantly intensifies soil loss beyond what would be expected from individual factors alone [45,46,68]. This highlights the need to integrate watershed-scale conservation planning with infrastructure protection strategies.

Overall, this study advances understanding of soil erosion dynamics in the Wolaita Zone by providing a spatially explicit, multi-factor analysis that integrates both natural and anthropogenic drivers. The findings demonstrate that erosion risk is highly localized and driven by the convergence of rainfall intensity, slope characteristics, soil properties, and land management practices. From a policy and management perspective, the results offer a clear basis for prioritizing intervention areas, particularly in high-risk zones where integrated conservation measures, including terracing, agroforestry, vegetative buffers, and improved land management, can substantially reduce soil loss. More broadly, the study reinforces the need for adaptive, site-specific, and watershed-based approaches to soil conservation to enhance land productivity, support climate resilience, and ensure long-term environmental sustainability [102].

### Limitations and overviews of soil erosion estimates with similar regional studies

The application of the RUSLE in the present study area provides a useful first approximation of soil erosion risk; however, several limitations and uncertainties must be acknowledged. The study relies on global and continental-scale datasets, including FAO DSMW soil data, NASA POWER rainfall, and ESA WorldCover LULC, due to the limited availability of high-resolution local datasets. While these products are widely used and validated, their spatial resolution and generalized nature introduce uncertainty. The coarse resolution and generalized soil units of the FAO DSMW dataset may not capture local-scale variability in soil properties, introducing uncertainty in erosion estimates. Accordingly, results are interpreted in terms of relative patterns and hotspot identification rather than precise absolute estimates. RUSLE is an empirical model originally developed under US environmental conditions, and its factors are often derived from generalized equations or literature values rather than being calibrated for local conditions. This can reduce its accuracy in complex topographies, where diverse agroecological systems and land management practices prevail. Furthermore, RUSLE primarily captures sheet and rill erosion but does not account for gully erosion, streambank erosion, or landslides, all of which are significant contributors to soil loss in the steep slopes. Potential error margins primarily arise from the resolution of input data and from parameter estimation. DEM-derived LS factors can vary across resampling, whereas K, C, and P values from global datasets may differ from local conditions. Additionally, R factors based on long-term coarse satellite rainfall estimates may lead to under- or overestimation of erosivity.

Due to the absence of ground-based measurements or sediment yield data in the study area, model outputs were validated using a comparative approach, drawing on published literature and case studies from ecologically and topographically similar regions in Ethiopia (Table 6). In addition to numerical validation, the spatial distribution of erosion-prone areas observed in this study aligns with topographic and land-use patterns typically associated with high erosion risk, such as steep slopes, degraded farmland, and areas with sparse vegetation cover. While the estimated mean soil loss rates in this study fall within the range reported for other Ethiopian watersheds (Table 6), notable differences can be attributed to variations in biophysical conditions, land use, and rainfall intensity. For instance, higher erosion rates reported in watersheds such as the Omo-Gibe and Rib watersheds [45,72] may be linked to steeper topographic gradients, higher rainfall intensity, and longer histories of land degradation. In contrast, relatively lower average values observed in the Gununo and Tikur Wuha watersheds [83,87] may reflect better vegetation cover, less intensive cultivation, or the presence of conservation measures that reduce runoff and soil detachment. Overall, the similarity in both magnitude and spatial pattern of estimated soil loss with those reported in peer-reviewed Ethiopian case studies supports the reliability of the current model outputs in the absence of ground-truth measurements. Nonetheless, future studies incorporating field-based erosion plots or sediment yield data are recommended to enhance the accuracy and calibration of the RUSLE model.

**Table 6:** Overview of estimated erosion rates for several locations in Ethiopia.

Source	Study area (ha)	Estimated erosion (tons ha^-1^ year^-1^)		Total annual soil loss (tons year^-1^)
		Range	Average	
[103]	Dembecha district (82,931)	0.02–291	49.00	526,996
[104]	Gununo watershed (20,440.89)	0–359.99	22.31	
[105]	Omo-Gibe River basin	0–279	69	89.6 million
[46]	Gumara watershed (20,440)	0.01–442.92	42.67	9.68 million
[45]	Rib watershed (197,500)	0 - 807	68	
[84]	Beshillo catchment (23,970)	0 - 935	37	
[68]	Gidabo watershed (330,200)	0–88.4	44.21	
[106]	Koga watershed (28,000)	0–265	47	255,283
[47]	Megech sub-basin (80,757)		41.54	843,736
[85]	Geleda watershed (25,609)	0 - 237	23.7	157,022
[107]	Upper Blue Nile Basin (292,230)		37.89	1.7 million
[108]	Gobele watershed (237,786.44)		34.26	1.02 million
[109]	Lake Hawasa Watershed (130,294)	0–262.48	28.2	27,783.4
[110]	Tikur Wuha watershed (68,100)		14.13	962,083
Present study	Wolaita Zone (450,900)	0.18–486	24.69	

## Conclusion

The present study was conducted in the Wolaita Zone, Ethiopia, to analyze soil erosion rates (tons ha^-1^ year^-1^) and map erosion-vulnerable areas by integrating the RUSLE model in ArcGIS. The Zone is highly prone to soil erosion due to steep terrain, variable rainfall, and diverse agroecological conditions, with risks further intensified by population pressure, intensive land use, and hydrological connectivity to downstream systems such as the Gilgel Gibe III Dam and Lake Abaya. The findings demonstrated the effective integration of the RUSLE model with GIS techniques to assess and quantify soil loss, identify erosion hotspots, map soil erosion risk across the study region, and provide spatially explicit evidence to support soil and water conservation measures. The southwestern and northwestern portions of the study region exhibited higher annual soil loss rates, ranging from 46.09 to 486 tons ha^-1^ year^-1^, accounting for 16.85%. The spatial variability in erosion intensity is influenced primarily by rainfall, steep slopes, soil type, and degraded vegetation cover. The north and eastern parts experience a low annual soil erosion rate (0.18 to 9.72 tons ha^-1^ year^-1^), due to substantial vegetation cover, good support practices, effective cover management, lower slope lengths, and gentle areas, covering an area of 171,054.45 ha (38.06%). The outcomes of this study can serve as a helpful tool for policymakers, land managers, and environmental planners to prioritize soil and watershed conservation efforts and implement management practices. Prioritizing areas with severe erosion risk for immediate intervention is crucial, including implementing comprehensive restoration strategies such as constructing physical structures, participatory watershed planning, terracing, stone/earthen bunds, and check dams, as well as intensive mulching. In very high erosion risk zones, conservation practices such as contour farming, strip cropping, Fanya juu/soil bunds, mulching, residue retention, and reforestation are recommended to slow flow velocity, and vegetative measures reduce detachment and enhance root reinforcement. For areas with a high erosion risk level, biological soil and water conservation practices are recommended, including vegetative buffer strips, agroforestry, cover cropping, and controlled grazing. These practices may reduce runoff velocity, increase infiltration, enhance soil organic matter, stabilize soil with root systems, and provide continuous ground cover that protects against erosion. In moderate erosion risk areas, crop rotation, conservation tillage, improved fallowing, and mixed cropping systems are suitable for maintaining soil structure, enhancing aggregate stability, and reducing detachment during moderate rainfall events. In low soil erosion risk zones, sustainable conservation practices are recommended, including minimum tillage, rotational grazing, cover cropping, and the preservation of vegetative buffer zones around farmland and water bodies. Ultimately, this study underscores the urgent need for integrated, multi-scale watershed management frameworks that combine geospatial analysis, local knowledge, and policy support. Future researchers may utilize a combination of RUSLE with more process-based models, such as WEPP (Water Erosion Prediction Project) or SWAT (Soil and Water Assessment Tool), to gain a more comprehensive understanding of sediment dynamics, particularly in complex terrains.

## Supporting information

S1 File POWER_Regional_Monthly_1984_2024_Average. (CSV)
